# Sex differences in knowledge and practices regarding antibiotics and antibiotic resistance in the Puerto Rican population

**DOI:** 10.1017/ash.2023.243

**Published:** 2023-09-29

**Authors:** Yiana Toro-Garay, Tanialy Rivera-Santiago, Vilmarie Ortiz-Bonilla

## Abstract

**Background:** Antibiotic resistance is one of the biggest threats to global health, and by 2050 it is expected to cause 10 million deaths per year globally. Sex differences depend on context and sociodemographic factors; therefore, studies addressing sex differences have been inconclusive. Furthermore, to our knowledge, sex differences in the Puerto Rican population have not been analyzed. We sought to understand whether knowledge and practices regarding antibiotic use and antibiotic resistance in the Puerto Rican population differ by sex. **Methods:** A convenience sampling was performed at outpatient clinics across Puerto Rico. Those who agreed to participate completed a self-report questionnaire aimed to address demographics, antibiotic knowledge, and experiences. Bivariate analyses were performed using Stata version 17.0 software. **Results:** In total, 252 participants received the questionnaire, and 250 completed it. Most of the participants were female (71.2%), aged >56 years (40.0%), and had a high school diploma (40.4%). Women had 2.71 (95% CI, 1.1–6.8, *P*). **Conclusions:** Women perceived themselves to be more knowledgeable regarding antibiotic use and resistance than men. However, no difference in actual knowledge could be identified. Similarly, antibiotic-related practices did not differ by sex except for using previously saved antibiotic treatment, and men had higher odds of conducting this practice. Further studies should be conducted to understand the factors that influence these behavioral practices, and educational interventions should focus on addressing misconceptions regarding antibiotics and antibiotic resistance.

**Figure:**
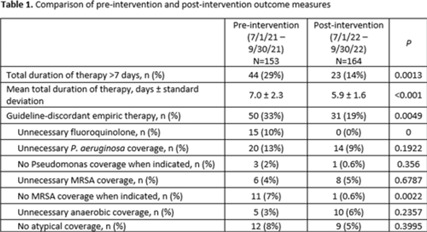

**Disclosures:** None

